# Learning clinical skills: an ecological perspective

**DOI:** 10.1007/s10459-022-10115-9

**Published:** 2022-06-24

**Authors:** Eric Brymer, Robert D. Schweitzer

**Affiliations:** 1 Faculty of Health, Southern Cross University, Gold Coast, Australia; 2 School of Psychology and Counselling, Queensland University of Technology, Brisbane, Australia

**Keywords:** Clinical psychology, Clinical training, Ecological validity, Learning design

## Abstract

The pedagogy underpinning clinical psychology training is often reliant upon the acquisition and transmission of knowledge and the practice of skills. The dominant paradigm in the training of clinical psychologists emphasises competence-based training drawing upon a scientist practitioner model of practice, often underpinned by knowledge of evidence-based interventions. Little has changed over the past 40 years. Training is predicated upon the assumption that effective therapy is attributed to the therapist’s skills to implement specific therapeutic processes and her or his capacity to form an effective working alliance with the client or patient. We provide an argument for an alternative paradigm in which ecological principles are privileged with a view to enhancing clinical training of psychologists in health settings responsive to the trainee as well as the broader societal context in which they practice, by adopting a pedagogy which prioritizes the relationship between the person and the environment. The proposed approach brings an ecological set of assumptions to the learning experience in clinical contexts. Key principles, drawn from an ecological perspective includes: affordances, the emergence of self-organisation in clinical learning, constraints and rate limiters. The approach is supported by examples applied to clinical learning contexts. Implications for clinical training are discussed. The ways in which an ecological approach may contribute to more effective learning outcomes through the use of representative learning contexts may inform learning design, how learning is actioned in clinical psychology as well as future research on the pedagogy of clinical training.

*I never teach my students I only attempt to provide the conditions in which they can learn*

Albert Einstein

The primary aim of this paper is to enhance understanding of the learning process in clinical education, by introducing an ecological perspective to understanding human behaviour founded on principles of Gibsonian (Lobo et al., 2018) ecological psychology, blended with dynamical systems theory (often referred to as Ecological Dynamics) (Alberto et al., In Press; Heft, 2012; Renshaw et al., 2009; Seifert et al., 2017). An ecological theoretical rationale based on Gibsonian ecological psychology and dynamical system theory provides guidance on how to design learning experiences with the environment in mind. This ecological approach proposes that individual and group behaviour is predicated on the person-environment relationship and the capacity to perceive and act on opportunities in the environment is fundamental to behaviour and learning. That is the environment plays an important role in behaviour and learning environments should form the focus of psychological and educational programs because they are linked in a direct way. A functional relationship between an individual and a performance environment can emerge through constant interactions by system components (e.g. individuals in a learning programme). The ecological approach to learning (e.g., Gibson & Gibson, 1955; Gibson & Pick, 2000) originated as an alternative to enrichment theories of learning. In enrichment theories the emergence of expertise with learning and experience is believed to occur through an increase in the sophistication of enriched individual internalized processes and structures (Jacobs & Michaels, 2007).

We demonstrate ways in which this approach may be applied to clinical learning contexts with a particular focus on clinical psychology. An important emphasis of this ecological approach to learning design concerns the relationship between the learner and the learning environment and the role of constraints on emergence of behaviors (Brymer & Davids, 2014). For these reasons we propose that an ecological approach to learning represents a viable framework for understanding the learning process and for designing learning opportunities in clinical education. Principles of the ecological approach are critical since the relationship between the individual and the learning environment defines this theoretical approach (Lobo et al., 2018; Meagher, 2020). Adding to the traditional scientist-practitioner model, in which the tenets of empiricism are prioritised, this ecological model appreciates that learning also emerges from the interaction between the learner and the physical and psycho-social environment (Brymer & Davids, 2014). This ecological approach to learning has been examined in sport (Araújo et al., 2006; Woods et al., 2020), psychology (Jacobs & Michaels, 2007; Lobo et al., 2018), education (Abrahamson & Abdu, 2020; Abrahamson & Sánchez-García, 2016; Brymer & Davids, 2013, 2014; Sharma-Brymer et al., 2018; Van Lier, 1997), information technology (Davids et al., 1991), health (Brymer et al., 2020), arts (Leduc, 2013), music (Schiavio & Kimmel, 2021), medicine (Onyura et al., 2015; van der Niet, 2018) and even mineral exploration (Davies et al., 2021). Individual-environment relationships are reciprocal in that the learner also impacts on the environment. In this paper we focus on the ways in which the characteristics of the person-environment relationship shape learning and how individual learners emerge as part of the environment for other learners. Key principles, drawn from the ecological perspective include affordances, the emergence of self-organisation in clinical learning, constraints, representative design and rate limiters.

## Traditional clinical education

Traditionally, clinical education in mental health has adopted methods which have been implemented across a range of performance contexts, often drawing upon the training of medical professionals, psychologists, nurses, and related professionals. For example, the contemporary model of education in graduate, applied psychology continues to be informed by a report dating back to 1941 and the development of the Boulder Model, named after a conference held in Boulder in 1949, which sought to establish a strong scientific foundation for psychology and the education of psychologists. An agreed upon set of standards for the training of clinical psychologists, which came to be known as the scientist-practitioner model was developed (Hayes et al., 1999). More recently, practitioners have argued for clinical education to be broadened to include a focus on preparing novice clinicians for (1) working with the needs of diverse people seeking assistance, (2) the interaction between psychologists and other health workers, and (3) taking a more critical stance in relation to a context often characterised by stigmatisation and marginalisation of people with mental health issues (Scheel et al., 2018).

In this paper we focus on learning design as it relates to the clinical education of psychologists in mental health settings. However, the principles underpinning this focus are applicable in clinical training more broadly. We argue that an ecological perspective provides greater ecological validity to learning experiences than models of education which foster “the educator” as expert, thus replicating the clinician as expert, independent of the field. That is, the relationship between educator, clinician and environment are considered. In contrast to perspectives that focus upon the idea that one size-fits-all, as highlighted by effectiveness in following set tasks (e.g. use of standardized interviewing, diagnosis, formulation and treatment planning), this ecological model recognises that learning is not an isolated mental process but stems from the relationship between the learner and the learning environment (Brymer & Davids, 2014). This is well exemplified in the research by Kraus et al. (2016) in which perceptions of the characteristics of therapists was shown to influence the response of patients to treatment programs independent of the treatment method provided. Significantly these influences are long lasting. Similar findings are reflected in our understanding of the therapeutic alliance and the ways in which the alliance is related to clinical effectiveness (Nienhuis et al., 2018).

The impact of this approach from a learning design perspective is that the notion of educators and tasks need to be reconceptualised for effective learning. However, the principles to be articulated have broader application across the full range of educational, developmental or therapeutic programs to encourage emergence of interpersonal characteristics such as personal responsibility, empowerment, creativity, teamwork, leadership, interdisciplinary collaboration, choice, reflexivity and personal growth. For a learning theory to be salient to the clinical education of psychologists in mental health settings, it needs to encompass a range of learning processes across a range of behavioural contexts.

## An ecological perspective on learning

From the ecological perspective explored in this paper the learner is considered to be an embodied, unique, complex system comprising many sub-systems which support thinking, perceiving, and acting (Davids et al., 2013). An educator recognises that each learner comes to the session with a unique set of intrinsic dynamics (dispositional characteristics) including physical, cultural, social, psychological and emotional influences that act as constraints on the acquisition of new skills, including the physical, emotional, psychological and social dimensions of those skills (Chow et al., 2011; Davids, et al., 2008). The learner will pass through periods of stability and instability reflected by varying levels of variability in behaviour as functional, goal-directed behaviours emerge. What this means is that each learner will find her/his own unique performance solution in his/her own time (Brymer & Davids, 2014).

Goal directed learning is recognised as a nonlinear process in the messy, noisy real world, characterised by progressions, regressions, jumps and skips, where each individual has to assemble her/his behavioural solutions from his/her intrinsic dynamics (Chow et al., 2007, 2011). From this viewpoint, learning clinical skills is a process of satisfying the unique interacting constraints impinging on each individual at any moment during his/her development. From a clinical educator perspective, it is, therefore, imperative to appreciate that each learner will begin the learning journey from a unique starting point, and follow an individual path stemming from the relationship between individual characteristics and the learning environment, and that learning is an open-ended process. For this reason, the time and approach taken by each individual to learn appropriate attitudes, qualities and behaviours may differ considerably, depending on the unique intrinsic dynamics that each learner brings to the task and the interacting constraints which shape each individual’s relatively unique responses.

### Individual, environmental and task constraints interact during learning

Constraints are key concepts in this ecological approach (Brymer & Davids, 2014). The word constraint is a technical term used to define the boundaries which shape the emergence of attitudes, qualities and behaviours during learning and performance. The interaction between constraints forces the learner to seek out stable and effective patterns of behaviour during goal-directed activity (Sánchez et al., 2016). A small change in one part of the learner-learning environment system can lead to large scale changes in behaviour emerging from particular patterns of interaction. This may lead to outcomes that support goal achievement or, if the learning context is ineffective, in some circumstances to outcomes that hinder goal achievement.

Newell (1986) classified constraints into three discrete groups: performer (individual), environment, and task. These notions will be partially illustrated by making reference to a key construct in fostering the development of clinical competencies in psychologists, that is, the concept of the therapeutic alliance. The reason for focussing upon the alliance is based upon the robust findings that the alliance predicts therapeutic outcomes, and probably accounts for more variance in therapy outcome than any other variable, including the type of the therapy, or the expertise of the therapist in particular therapy modalities (Baier et al., 2020; Horvath & Symonds, 1991). Constraints may be translated into a clinical teaching pedagogy by referring to individual constraints, environmental constraints, and task related constraints. This approach has been exemplified in the formulation of the alliance by Ross et al. (2008), who note the importance of conceptualising the alliance as relational involving the therapeutic environment, client characteristics and therapist characteristics as well as the therapeutic tasks undertaken.

*Individual constraints* are the unique structural and functional characteristics of each learner and include physical, psychological, cognitive and emotional factors. A learner’s genetic makeup, body shape and size, gender and racial identity, technical abilities, stage of development, past experiences and psychological factors like beliefs, anxiety and motivation and even relational trauma may shape the way individuals approach learning the tasks which underpin practice. This notion is very important for learning designers and facilitators because we cannot assume that all learners are starting from the same place, learners are different from each other and therefore a one-size-fits-all approach to learning design is unlikely to be effective for every learner. This approach highlights, accepts and responds to the unique features of each learning context. For example, while a fully trained practitioner’s capacity to access their own emotional responsiveness is critical to their capacity to engage, often referred to as being empathically attuned to the other with a view to gaining an understanding of the other, the capacity to develop the skills to perform effectively in this area will partially depend on how effective the student is at accessing their own emotional state as part of the learning process.

From an ecologically-informed learning design perspective the person-environment relationship provides possibilities or invitations for action, known as affordances, and plays a significant and important role in constraining (facilitating and limiting), or shaping the performance solution and style that emerges for each individual (Withagen et al., 2012). At the same time, the relationship is reciprocal and the learner brings their own predispositions, capacities and affect into the learning situation which influences the learning process. These different individual constraints support the distinct strategies that may be used to understand human relationships or solve interpersonal problems. Resolutions which emerge from the activities of different learners highlight the need to adopt a learner-centred approach in clinical learning contexts. These unique performer characteristics can be viewed as resources for each individual that channel the way in which each learner solves particular problems (e.g., how to behave in a manner that fosters the therapeutic alliance for a particular dyad) or characteristics that can lead to individual-specific adaptations (e.g., how best to manage physical characteristics such as height in a therapeutic context) (Brymer & Davids, 2014).

From a pedagogical perspective the ecological framework presented can be contrasted with a traditional scientist practitioner model in which evidence-based procedures are prioritised (Shedler, 2018). That is, this ecological model privileges adaptation and the learning which emerges from such adaptation, within the learning environment. For example, a cursory review of clinical training programs will identify an emphasis upon the models of therapy taught, such as Cognitive Behaviour Therapy (CBT) with little reference to context. Whereas training in a therapeutic technique such as CBT may specify the importance of undertaking specific techniques, such as challenging thoughts, an ecological perspective encourages the learning designer to also design environments and tasks that provide opportunities for the learner to experiment and consider the unique needs of the individual within their performance or clinical context. This does not mean that technique-oriented learning design is necessarily inappropriate, rather it asks the clinical educator to reflect carefully.

From the ecological perspective learning design as applied to the context of clinical psychology is partially about shaping environments and tasks that help leaners perceive and act upon affordances that would be important for effectiveness in the real-world context of promoting mental wellbeing (Young et al., 2020). Within the clinical context, such environments are invariably interpersonal, or relational, hence the imperative to adopt a relational stance in which both poles of the relationship are recognised. In addition, this will allow the learner to appreciate aspects of the leaning which are not easily explained but result from the vantage position of “observer” of the relationship, and the role of the relationship in learning. This point of vantage is sometimes referred to as “the third” in contemporary writings (Orange et al., 1999). That is, the learner will have embodied experiences which are critical to his or her learning but may remain part of a sensory- motor repertoire of understanding. Within the psychotherapy literature, the experience of, and making sense of counter transference (feelings experienced in the therapist whose origins may relate to the therapist’s own early relationships) would be an example of such learning.

Notions of task emerge in both a pedagogical context and in a clinical context. Personal constraints in the pedagogical context are not to be viewed as barriers to a perceived ideal. Indeed, an individual’s pragmatic solution to task challenges emerges to satisfy his/her own unique constraints. Variability in behaviour plays a functional role as each individual pursues a task goal in his/her own way (Davids et al., 1991). To an extent, learners in clinical psychology contexts familiar with more direct instructional methods of teaching and ideas of disorders as residing within an individual mind may struggle somewhat with the ecological framework. From a clinical educator perspective this idea indicates that educational techniques that expect a *prescribed* outcome might limit individual learning. Rather it is important to recognise that behaviour is emergent under constraints and that consistent performance outcomes can be achieved in a variety of different ways as learners satisfy the interacting constraints on them (Chow et al., 2011).

An example of important constraints in the clinical context include the expectations which are implicit in the learner and clinical educator and are brought to the clinical learning context, whether that relates to their conception of wellbeing, notions around diagnosis, or the meaning of effective interventions. This idea, of acknowledging the assumptions brought to clinical contexts, also suggests that the practice of modelling an assumed ideal behavioural template might conceivably serve to limit learning, unless it is used to help the learner move to the right ‘ball park’ of useful adaptive behaviours, rather than used to prescribe in advance a specific behaviour (Van Lier, 1997). This is often evidenced in the development of types of “teaching manuals” which emphasise a ‘one size fits all’ approach. From an ecological perspective modelling can only serve to guide learners towards finding a useful approximation of a potential outcome.

*Environmental constraints* are most often presented as physical and socio-cultural factors (Brymer & Davids, 2014). In a clinical learning context *physical factors* comprise the immediate surroundings and include the class set up, sounds, colours, and the information available in learning contexts (Steffy et al., 2017). Within clinical training programs, the capacity of clinics to incorporate video technology into training may be critical, however, care might be required for its effective use (Haggerty & Hilsenroth, 2011). *Socio-cultural* factors in a learning context include the role of peer groups, the clinical educator, and cultural expectations. Important socio-cultural environmental constraints include fellow learners, the presence of support and access to high quality learning facilities (e.g. affording safety, engagement, social interaction, reflective practice). Psychological safety, such as the learner’s perception that they can voice concerns in the learning context without feeling that this might adversely impact on their leaning outcomes or their relationship to the clinical educator is an example of a potentially impactful environmental constraint (Caverzagie et al., 2019). In clinical education the clinical educator is a particularly important environmental constraint on the learner and the skill, beliefs, fears, attitudes and style of an educator can have a positive or negative impact (rarely neutral) on educational outcomes, as well as important related issues such as clinical effectiveness ensuring the safety of people seeking assistance from trainees (public safety). Even perceptions about educator support can influence the learning outcomes. A common example relates to instructors often representing the dominant group of white, privileged, able-bodied, cis-gendered men and women. The “black lives matter” and also the “me too” campaigns addressing the need for greater awareness of implicit prejudice may play an increasing influence on the development of clinical psychologists demonstrating gender and multicultural competencies long ignored in mainstream education (Neville et al., 1996).

From a learning designer perspective, the learning environment is particularly important as the designer and facilitator need to have awareness of the values and behaviours which he or she may bring to the learning experience, a capacity to engage in reflective and reflexive critical thinking and demonstrate awareness and openness to alternative ways of being-in-the-world, which are often subtle and implicit. All of these issues need to be considered in grasping the influence of socio-cultural factors in the process of learning design. The requirement is challenging to learning designers who may at times experience a degree of discomfort, when asked to create learning contexts representative of the professional clinical world.

*Task constraints* consist of the goal of the specific task (such as creating the therapeutic alliance or undertaking a particular assessment while keeping the alliance in place), the learning location, conventions and goals of the activity and the implements or equipment used during the learning experience (Newell, 1986). In contrast to the other constraints the clinical educator is easily able to manipulate task constraints, for example deciding on teaching style or setting boundaries and goals. Small manipulations by an educator, for instance attending to boundary issues in clinical contexts, can often lead to large scale changes emerging in a learner’s behaviour, such as the emergence of a greater awareness of boundaries in clinical practice, thus enhancing the learning about safety of the public seeking clinical interventions.

In clinical education the task is often perceived by participants as integral to the power of the learning experience. For example, there is a significant literature on the role of trauma-informed care and the ways in which traditional institutions, and in particular, those which may incorporate processes such as seclusion and involuntary treatment regimes, are said to perpetuate rather than relieve the psychological strains associated with early trauma (e.g. Wesselmann & Parris, 2021). However, with greater awareness of the sensitivities required in trauma-sensitive care, including awareness of gender, race and socio-cultural factors, educators can be increasingly effective in manipulating task constraints with a view to promoting effective reflexive practice. Such practice will play a role in guiding learners to being more reflexive and also discovering helpful learning patterns and decision-making behaviours. Particularly if the constraints are *representative* of the intended performance context (Pinder et al., 2011). In practice, this may mean that the learner is placed in situations in which trauma episodes may be triggered, and be guided in developing a greater capacity to recognise those events which are likely to trigger trauma responses, and develop strategies for affect regulation, which may well be interpersonal. Such learning cannot be achieved through theory but requires the experience of dealing with acute affect which is evoked in particular clinical learning contexts.

Most commonly, the learning context is represented in psychology programs through training institutions providing clinical space within which the novice therapist is able to ‘practice the skills being taught’, in an environment allowing for video recordings and close clinical supervision (Haggerty & Hilsenroth, 2011). From the perspective being advocated, the degree to which client presentations represent the issues presented by clients in external clinical settings, the greater the ecological validity of the learning experience. That is, the relationship between the performance environment and the clinical education context is ‘representative design’ (Pinder et al., 2011). Representative design refers to the process of ensuring that the essential elements of the real-world context (e.g., the therapeutic context) are accurately represented in the learning environment. Learning design is constituted by both environment and task constraints. In the context of clinical education, this does not imply that the learning task *must* physically represent the real-world situation but that the information (e.g. social, emotional components of clinical relationships) should be accurately mirrored. This concept indicates that the clinical educator must possess a mastery of knowledge and experience in specific activities and a deep appreciation of the *relevant* information and capacities inherent in the learner’s life. An effective educator is able to use this knowledge and mastery to design experiences that effectively render the learning context a representation of the real world clinical context (Brymer & Davids, 2014). Thus, in the context of developing the therapeutic alliance the clinical educator will need to design and facilitate the learning environment and task (representative design) in a manner that is representative of the therapeutic alliance in the real world. It is not enough to talk about the topic or describe the mechanics of achieving tasks (such as empathy), the learning context must be representative in the sense that the learner is able to perceive appropriate opportunities and find their best way to facilitate the therapeutic alliance with others (this could be dependent in part on culture, physical size, gender, language, facial expressions, feelings of comfort and so on and in part the choice of colour or decorations in the clinical learning spaces, a window, the right furniture and even the right music in the reception space can impact on how a client prepares for the therapeutic process). For example, many if not all clinical training programs include a Psychology Clinic which provides students with their first exposure to working with the public. From an ecological perspective the very structure of such clinics serves to “model” a particular form of practice. In the Clinic where one of the authors supervises students, the first thing one notices in entering the consulting room is a large whiteboard, generally clean, and two chairs, a small table, a box of tissues and a discrete video camera. These are the most obvious “props”, which may convey some expectations and the capacity of the therapist to arrive at “explanations” and “downward arrows” which may be explained on the whiteboard, for those novice therapists who are approaching their work from a CBT perspective. A subtler process is the use of therapy feedback which is associated with effective clinical outcomes. Rather than the prop of the whiteboard, the trainee is encouraged to discard pens and paper, focussing instead, upon the relationship and those elements which have been associated with a good therapeutic alliance between therapist and patient.

### Emergence, self-organisation under constraints and rate limiters

As noted above, following the ecological framework presented here, effective behaviours *emerge* from the dynamic relationship between the various interacting constraints. Beneficial behaviour is neither completely predictable nor random. In learning tasks involving group work for example, skills that are required for effective social interactions as well as the enhancement of goal achievement emerge from the particular interactions between the specific inter-personal and intra-personal constraints as well as those inherent in the environment and task. However, as each learner enters the particular learning journey at his/her own unique point the learning process and problem resolutions will also be unique to each learner and even open-ended.

The ecological perspective proposes that learners’ responses are *emergent,* based on the interaction of various subcomponents (e.g. past social experiences, emotional readiness, ability to relate with a range of people from diverse backgrounds) and the ability of these subcomponents to self-organise or naturally adjust and adapt (Lobo et al., 2018). Learners achieve stable behavioural patterns based on the interaction between individual constraints and those inherent in the task and environment. The implication of this idea is that clinical educators need to design activities where the task and environment are conducive to the emergence of clinically relevant, intended learning outcomes. The challenge for each clinical educator is to help each learner find their own functional outcomes in satisfying the unique set of interacting constraints. However, the learning “space” will need to be representative of the professional clinical context for each learner. For example, if a clinical learning experience was designed as a medium to enhance capacities to develop the therapeutic alliance in a particular real-world professional context it will work best if the clinical learning experiences reflect the workplace context (Araújo et al., 2006; Pinder et al., 2011). A good example is found in the training of therapists to work with people with eating disorders, where there are complex interactions between practitioner values and competencies, and various internal and external pressures related to body image, cultural stereotypes, environmental contexts, the exercise of power and also medical concerns. As well as the specific requirements for providing opportunities for practitioners to enhance their skills in facilitating the therapeutic alliance (Zaitsoff et al., 2015) they need to develop skills in multidisciplinary team work comprising medical, psychology and dietetic specialists who are required to work collaboratively with the client. The development of interdisciplinary teamwork skills are complex. Effective learning design would include tasks representative of the real-world therapeutic process.

As discussed earlier, the ecological perspective highlights the recursive nature of the learning process. Clinical educators need to appreciate that the process of learning is often non-linear. For example, *rate limiters (in the sense of information that is restrictive to learning),* impacting upon the learning trajectory may include environmental, task or individual factors that limit the rate of learning and development in an individual (Brymer & Davids, 2014). Within the context of professional development, rate limiters might be linked to the developmental status of the learner and include emotional readiness of participants, physical characteristics such as height and body size (e.g., a more petite therapist with a physically large and dominating client or vice versa), the appropriateness of the clinical learning context, and even the clinical educator. This is most obvious in clinical learning when working with children but may be present in all such interactions.

A skilled clinical educator may manipulate constraints in such a way to mediate the effect of rate limiters that are most obviously hindering learning progressions. For this purpose, clinical educators need to be skilled at determining each individual’s needs in order to manipulate the specific task (or environmental) constraints to best draw out the intended learning process. For example, the use of group learning and feedback on interpersonal processes may provide a unique opportunity for learners to gain greater self-knowledge of the various ways in which they express themselves, or use their height, expertise or seating position, impact upon a particular client. Ineffective attempts to design and facilitate learning environments and tasks could inadvertently provide cues or be representative of a learning context that shapes rigid, uncertain, critical, tense, and distracted learners who over structure therapy sessions, and use silence and self-disclosure inappropriately (Ackerman, & Hilsenroth, 2001).

### Affordances

As briefly noted above affordances, as these relate to the development of a more responsive clinical pedagogy, are opportunities for action that emerge from the relationship between the individual and the environment. The affordance notion is directly related to a fundamental aspect of Gibsonian ecological psychology, direct perception. From this perspective all animals (including humans) perceive the environment directly in terms of the possibilities for actions. This is often compared to more traditional frameworks which assume an indirect perception which relies on the development of internal representations (van Dijk & Kiverstein, 2021). The notion of affordances explains how perceptual capabilities can guide behaviour without requiring a conscious interpretation of internally augmented representations.

The affordances of the environment are what it offers the animal, what it provides or furnishes, either for good or ill. The verb to afford is found in the dictionary, but the noun affordance is not. I have made it up. I mean by it something that refers to both the environment and the animal in a way that no existing term does. It implies the complementarity of the animal and the environment (Gibson, 1979, p. 127).

What this means is that a given learning environment will be comprised of specific properties which provide opportunities for action from a learner’s own unique perspective. Conceptually, this changes the typical perspective on the environment from what the environment looks like to one where the environment can be described in terms of action possibilities (Chemero, 2003; Withagen et al., 2012). A surface or ledge in an environment may be ‘jumpable’ or ‘step-on-able’ for different learners (Heft, 1989). In real-world clinical contexts types of chairs might suggest sitting, lounging or lying down. Equally colours of walls or sounds might afford relaxation, feelings of joy or feelings of sadness. Similarly, individual learners will perceive opportunities for independent work or for emotional development in the same learning environment. Learners need to be placed in representative learning environments where they can attune to implicit and explicit information enabling them to make real-world relevant and informed decisions based on a complete understanding of their own capabilities in any given environment. Affordances may change as a function of time and context, for instance with development, experience and expertise in a specific task.

### Perception-action coupling and representative learning contexts

From an ecological perspective, a learner will only develop effective real-world relevant skills and capacities if learning takes place within an appropriate representative learning context. What this means is that a learner must first perceive and interpret information that is relevant to the real-world scenario, in turn this will lead to real-world relevant actions for task goal achievement. It is for this reason, that learning will benefit from being based in a clinical context comprising experienced clinicians, clients and learners, thus representing an authentic learning experience. This perception–action mutuality has a cyclical structure, and this coupling is strengthened through a process of *attunement* to relevant information in the learning environment to support real-world actions (Bruineberg et al., 2021). In effect for useful learning to take place the clinical learning context must be representative of the everyday clinical context. Reducing an intended behaviour to decomposed parts and practicing an activity out of context breaks up this perception–action coupling. The result is an unproductive development of less functional behaviours that may not fit the performance context since learners are not gaining experience at picking up relevant information from a specific performance context and using it to support behaviour.

## The ecological approach in the context of clinical education

Manipulating task constraints is the simplest way to change learners’ behaviours from this theoretical viewpoint. This process can be as simple as ensuring that if a program has been sponsored to facilitate building the therapeutic alliance then the activities are also designed to represent the development of a therapeutic alliance. Equally, constraint manipulation might also occur during an activity, for example when scenarios are adjusted or restrictions added to guide learners towards specific experiences, or boundaries adjusted during group-based learning experiences. However, task manipulation does have pitfalls for the inexperienced clinical educator if constraints are manipulated without careful consideration. As noted above ineffective tweaks can inadvertently be representative of ineffective behaviours such as rigidity. Such a strong theoretical framework can help unskilled clinical educators avoid creating artificial task constraints which restrict rather than support the emergence of intended outcomes and behaviours. As noted earlier, constraint manipulation must be based on the key pillar of task representativeness in order to provide the opportunity for learners to attune to key affordances for action which can develop relevant real-world behaviours in individuals. Setting appropriate challenges for learners can be stimulating and depends on the clinical educator’s capacity to identify the key factors a learner needs to work on at any specific stage of his/her development. Clinical educators need to be able to identify whether key constraints will enhance the intended learning process or act as a “rate limiter” to the acquisition of real-world relevant skills and behaviours. This confirmation process needs to be ongoing as task constraints are dynamic and can emerge and decay over time (Guerin & Kunkle, 2004). From an ecological perspective, effective manipulation of task constraints is dependent on individual, group, environmental and time contexts.

Learning is a holistic journey centred around the key processes of perception, cognition, reflection and action. The interplay between the learner, the clinical educator, the social context, the physical environment are considered essential to the learning process. These concepts are recognised by the ecological perspective because the learner is considered central to the experience and the interaction between the learner and the environment is a key relationship in the learning process. Perception, action and reflection are of paramount importance and need to be understood with respect to the learner-environment relationship. The ecological perspective acknowledges that learning is not an isolated mental process and cannot be separated from the context since it emphasises the importance of providing experiences, as distinct from instructions, as a means of facilitating learning.

From this viewpoint learning does not need to be confined to a linear (or cyclical) stage model, but rather is dynamic and unpredictable. This does not mean that a clinical educator cannot or indeed should not carefully design and plan to facilitate an experience or to engage with predetermined learning exercises. Rather, the approach being proposed places more emphasis on the appropriate learning design and facilitation of clinical activities. From an ecological perspective it may not be ideal to select an activity from a book and fit it to an audience. The activity needs to be designed to fit each specific learning context and “real word” requirements. Strategies to achieve optimum learning design are presented in the section following.

## Learning strategies in ecological learning design

From an ecological framework perspective, learning design process is key to behavioural outcomes and effective learning facilitation is dependent on several key strategies. First, the ideas of affordances and task representativeness suggest that it is important to ensure that the learning design truly reflects the real-world context. Preparing a group of professional psychologists to undertake a clinical education activity must be carefully planned to accurately represent the relevant aspects of the natural performance requirements. The clinical educator needs to carefully assess which information sources are vital for effective learning and bring these aspects to the clinical learning context. For example, the ecological approach suggests that the social environment should represent the real-world context. This concept might also explain variability in terms of effectiveness of transferability from the learning context to the clinical context (van den Ertwegh et al., 2013). Equally the concept of task representativeness suggests that a clinical educator will need to determine the real-world performance constraints in the therapy context before she/he can design a truly effective program. This is quite different to the paradigm which emphasises evidence-based practice as if it equates to a reductionism and a focus upon “method”, whether that be CBT or other therapeutic model.

Second, the idea that learning is emergent and that a learner self-organises (self-adjusts and adapts) under constraints indicates that the clinical educator cannot presuppose that there will be a specific outcome to a particular problem from each individual. Each learner will start the learning journey from their own unique place and take their own unique path. Education design and implementation should not be geared towards each learner using the same putative optimal strategies in each context in a ‘one-size-fits-all’ approach. It is much more learner-centred and the clinical educator’s task is to help each learner consolidate his/her learning. The challenge is for each clinical educator to take responsibility for ensuring that the clinical task truly represents the learner’s needs and that constraint manipulations are relevant and functional to lead to specific, intended behaviours. How these behaviours emerge may vary considerably in each learner. The clinical educator must develop the communication and learning design skills to ensure they remain learner-centred throughout the learning experience. In practice, this can often be achieved through individual supervision of clinical work, undertaken by the learner, in a context where the educator provides a safe space so the learner may feel free to discuss his or her vulnerabilities and at the same time, the educator is able to address the learner’s strengths and areas for enhancing practice. The educator needs to be able to guide the leaner to achieve a learning outcome (e.g. developing a safe and constructive alliance with different clients from different backgrounds) in a manner appropriate for the learner.

The third key strategy revolves around the concept of rate limiters. A clinical educator needs to have the knowledge and skill to also determine what aspects might be limiting a learner’s ability to acquire particular attributes or behaviours (including the capacity to realise that the educator’s own style, behaviours, values and beliefs might be the greatest rate limiter). This is not based on a predetermined understanding of specific behaviours but on the context and individual in question. For example, in learning the assessment and diagnostic skills which underpin clinical psychology, it might be that the task problem starts to overshadow the intention to facilitate an activity where learners were to be encouraged to think critically about diagnosis. A learner might want to administer a diagnostic instrument or symptom inventory, such as the Beck Depression Inventory, to inform them in arriving at a diagnosis of depression. In the process, they would be missing the context in which the person was living and the influence of power relationships, say, within a coercive interpersonal relationship. In this instance, it might not be appropriate for a skilled clinical educator to wait for the learning experience to unfold and facilitate a group discussion. It might be more appropriate to manipulate task constraints in order to rekindle the intended skill acquisition.

## Summary and implications

We have argued for a shift in pedagogical models as applied to the learning of clinical skills. Drawing upon the training of professional psychologists as an exemplar for understanding the proposed shift from a more traditional “expert-based” scientist practitioner orientation to an ecological paradigm drawing upon Gibsonian ecological psychology,.with a view to guiding educational practice in terms of its capacity to be responsive to the person-environment context The ecological model being proposed emphasizes the learner as a complex system and that learning emerges through important interactions of individual, environmental and task constraints. Since the first two categories are unique to individual learners, it follows that variability in learning solutions should be expected by clinical educators. From an ecological perspective, clinical educators planning to design effective learning experiences may need to conceptualise the unique task and environmental constraints that frame a particular learner and ensure that the learning task is representative of the real-world performance contexts relevant to providing optimum learning experiences for people in clinical psychology.

From a clinical education perspective the challenge of the ecological approach are that it requires clinical educators to (1) ascertain the vital information sources appropriate for a particular learner in a specific clinical learning context, (2) design activities that effectively utilise this information, (3) understand the ongoing individual and environmental dynamics and readiness and (4) incorporate these into program design. The clinical educator’s ultimate responsibility is to ensure task and environment constraints are manipulated to enhance ecologically valid learning as applied to the different dimensions of humans as complex systems. The potential outcome will be practitioners who are not only versed in the theory which underpins their competencies, but a new skill set that emerges in the context of their community, considering the nuances posed by particular personal, social and cultural constraints.
